# Prognostic Role of Lesion Morphology in Patellar Osteochondral Fixation: Influence of Localization and Size on Functional and Pain Outcomes

**DOI:** 10.3390/jcm14248829

**Published:** 2025-12-13

**Authors:** Yunus Emre Bulum, Abdullah Burak Kara, Muhammed Enes Karataş, Mehmet Mete Oruç, Abdullah Demirtaş, Oğuz Şükrü Poyanlı

**Affiliations:** Department of Orthopaedics and Traumatology, Göztepe Prof. Dr. Süleyman Yalçın City Hospital, Istanbul 34010, Türkiye

**Keywords:** patellar dislocation, osteochondral lesion, fixation, lesion localization, trochlear groove, clinical outcomes

## Abstract

**Background/Objectives**: Osteochondral lesions are common after acute patellar dislocation, particularly in young, active individuals. Although fixation of osteochondral fragments is a preferred treatment to restore joint congruence and prevent cartilage degeneration, the prognostic effect of lesion localization and size on postoperative outcomes remains unclear. **Methods**: This retrospective clinical study included 75 patients (mean age 23.8 ± 6.1 years) who underwent osteochondral fixation after patellar dislocation between 2018 and 2024. Lesions were classified by location—medial patellar facet, lateral facet, trochlear groove/lateral femoral condyle, or multiple regions—and measured on preoperative MRI for size and depth. Functional outcomes were assessed using Kujala, Lysholm, Tegner, and VAS pain scores preoperatively and 12 months postoperatively. Associations between lesion characteristics and postoperative results were analyzed using *t*-tests, ANOVA, and Pearson correlation. **Results**: Lesions were most frequently located on the medial patellar facet (42.7%), followed by the trochlear groove/lateral femoral condyle (34.7%). Larger and deeper lesions were more common in trochlear or multiple-site involvement (*p* = 0.002 and *p* = 0.031, respectively). At final follow-up, all functional scores improved significantly (Kujala: 62.4 ± 11.3 to 86.7 ± 9.1; Lysholm: 64.1 ± 12.0 to 88.2 ± 8.3; VAS: 6.2 ± 1.3 to 2.1 ± 1.1; all *p* < 0.001). Patients with smaller lesions (<100 mm^2^) and medial facet involvement achieved higher Kujala scores (*p* = 0.020) and lower postoperative pain (*p* = 0.001). The overall return-to-sport rate was 78.7%, and postoperative complications occurred in 10.7% of cases, mainly recurrent dislocation or implant irritation. **Conclusions**: Lesion localization and size significantly affect postoperative outcomes after osteochondral fixation for patellar dislocation. Medial facet and smaller lesions are associated with better pain relief and functional recovery, whereas large or trochlear lesions carry a less favorable prognosis. Morphological assessment of lesion characteristics should therefore guide surgical planning, fixation strategy, and postoperative rehabilitation to optimize clinical outcomes.

## 1. Introduction

The patellofemoral joint plays a critical role in knee stability and is particularly prone to dislocation in young and physically active individuals. Acute patellar dislocation is frequently accompanied by osteochondral injury, with reported incidences ranging from 30% to 70% in the literature [1,2,3]. These lesions often necessitate surgical intervention, as unrecognized or inadequately treated osteochondral defects may lead to early cartilage degeneration, chronic pain, and functional impairment [4,5,6].

Osteochondral fragment fixation has become the preferred treatment strategy to preserve joint biomechanics and prevent progressive degenerative changes. Anatomical reduction and stable fixation with metallic or bioabsorbable implants have been associated with favorable outcomes and early mobilization [7,8,9]. However, various prognostic factors have been proposed to influence clinical results following fixation, including patient age, trauma mechanism, concomitant ligament pathology, rehabilitation protocol, fixation type, and, most notably, the localization and size of the chondral lesion [10,11].

The medial patellar facet is the most frequently affected region; however, lesions involving the trochlear groove or lateral femoral condyle tend to present with larger defect areas and more complex biomechanical implications [12,13]. Previous studies have suggested that increased lesion size and depth are associated with reduced fixation stability and inferior postoperative functional outcomes [14,15,16]. Nevertheless, evidence regarding the relationship between lesion morphology and surgical prognosis remains limited and somewhat controversial.

Importantly, lesion morphology is expected to influence prognosis not only due to variations in defect size but also because of fundamental biomechanical and biological differences across anatomical sites. The trochlear groove is subjected to higher compressive and shear forces during mid-flexion, has a distinct cartilage thickness profile, and demonstrates less favorable load distribution than the medial patellar facet [17,18]. These factors may impair osteochondral fragment stability, limit revascularization potential, and increase susceptibility to residual pain or early degenerative changes. Similarly, larger defects disrupt joint congruence and focal load transmission, increasing contact pressures and reducing the likelihood of durable fixation [18,19].

Despite these well-recognized biomechanical considerations, prior studies examining lesion morphology after patellar dislocation have often been limited by small sample sizes, heterogeneous patient groups, and inconsistent MRI-based measurement techniques [20]. As a result, reported associations between lesion localization, lesion size, and postoperative prognosis have remained inconclusive, underscoring the need for larger cohorts with standardized evaluation. The present study specifically addresses this gap by analyzing a broader and clinically diverse population, enabling a more reliable assessment of morphological predictors [20,21].

Therefore, the present study aimed to evaluate the relationship between lesion localization and size and postoperative clinical outcomes in patients who underwent osteochondral fixation following patellar dislocation. Additionally, the potential influence of different fixation techniques and concomitant surgical procedures on postoperative recovery was analyzed. This study seeks to contribute to a better understanding of the morphological factors affecting prognosis in the surgical management of osteochondral lesions of the patellofemoral joint.

## 2. Materials and Methods

### 2.1. Study Design and Ethical Approval

This study was designed as a retrospective clinical investigation. Medical and imaging records of patients who underwent osteochondral fixation after patellar dislocation between 2018 and 2024 at the Department of Orthopaedics and Traumatology, Göztepe Prof. Dr. Süleyman Yalçın City Hospital, were reviewed. Ethical approval was obtained from the hospital’s Non-Interventional Clinical Research Ethics Committee (Decision No: 2025/0181, Date: 25 September 2025). The study was conducted in accordance with the Declaration of Helsinki, and patient data confidentiality was strictly protected.

### 2.2. Patient Selection

A total of 75 patients were included in the analysis. Inclusion criteria were: (1) age between 15 and 40 years, (2) surgical treatment with osteochondral fixation after primary or recurrent patellar dislocation, (3) complete preoperative and postoperative (12-month) clinical data available, and (4) a minimum follow-up period of 12 months. Patients with multi-ligament injuries, prior knee surgery, advanced degenerative changes, or incomplete follow-up were excluded. All data were verified through operative notes, outpatient records, and radiological archives.

### 2.3. Surgical Technique

All procedures were performed by experienced orthopedic surgeons using a standardized approach. After joint exploration, osteochondral fragments were identified, anatomically reduced, and fixed to their original bed. Fixation was achieved with metallic screws, bioabsorbable screws, or smooth Kirschner wires, selected according to fragment size, localization, and bone quality.

To minimize variability, all surgeons followed the same institutional surgical protocol, including arthrotomy exposure, fragment preparation, and fixation sequence. Although implant type (metallic vs. bioabsorbable vs. K-wire) was selected according to fragment characteristics, this choice was made within predefined institutional guidelines and did not reflect surgeon preference. Therefore, the operative technique was largely homogeneous across the cohort, reducing potential procedural confounding.

In patients with maltracking or instability, adjunctive realignment procedures such as lateral retinacular release or tibial tubercle transfer (TTT) were performed according to standardized indications.

### 2.4. Postoperative Rehabilitation

Postoperatively, all patients underwent a uniform rehabilitation protocol.

The knee was immobilized in extension for the first two weeks, with controlled passive motion initiated in the first postoperative week (0–60°). Partial weight-bearing of approximately 20–30% body weight was permitted after the third week, progressing to full weight-bearing by weeks 5–6, provided that pain and swelling were controlled. Strengthening focused on closed-chain quadriceps activation, hip abductor strengthening, progressive core stabilization, and neuromuscular/proprioceptive exercises while avoiding excessive patellofemoral loading during early recovery.

Return to sport was permitted after the completion of functional recovery, generally within 9–12 months.

### 2.5. Clinical and Radiological Evaluation

Demographic data (age, sex, affected side, body mass index) and clinical parameters (injury mechanism, time interval from dislocation to surgery, and previous dislocation episodes) were recorded. Lesion localization was determined from operative and MRI reports and categorized as medial patellar facet, lateral facet, trochlear groove/lateral femoral condyle, or multiple regions. Lesion size (mm^2^) and depth (mm) were measured from preoperative MRI using multiplanar reconstruction.

All MRI-based measurements were performed independently by two musculoskeletal radiologists with over 8–10 years of experience. To assess measurement reliability, lesion size and depth were re-evaluated in 20 randomly selected patients. Intraobserver agreement was excellent (ICC = 0.92), and interobserver agreement was high (ICC = 0.88), indicating strong reproducibility of the imaging-based measurements.

Functional outcomes were assessed using the Kujala Anterior Knee Pain Scale, Lysholm Knee Scoring Scale, Tegner Activity Level Scale, and Visual Analog Scale (VAS) for pain. Each patient was evaluated twice: preoperatively and at the 12-month postoperative follow-up. Additionally, the time to return to sport (months) and postoperative complications (recurrent dislocation, hardware irritation, arthrofibrosis, nonunion) were documented.

### 2.6. Follow-Up Protocol

All patients were examined clinically and radiologically at the final 12-month follow-up. Functional scores were compared with their preoperative values. Radiographs and MRI, when available, were reviewed to confirm fragment union, implant integrity, and joint congruence. Patients with clinical suspicion of redislocation underwent additional imaging evaluation.

### 2.7. Statistical Analysis

All statistical analyses were performed using IBM SPSS Statistics for Windows, version 26.0 (IBM Corp., Armonk, NY, USA). The normality of distribution was assessed using the Shapiro–Wilk test. Continuous variables were presented as mean ± standard deviation (SD), and categorical variables as frequencies and percentages. Comparisons between categorical variables were conducted using the chi-square test or Fisher’s exact test where appropriate. For normally distributed continuous variables, independent sample *t*-tests were used for two-group comparisons, and one-way analysis of variance (ANOVA) was applied for comparisons among more than two groups. When ANOVA results were significant, Bonferroni correction was applied for post hoc pairwise analyses. Non-parametric equivalents (Mann–Whitney U or Kruskal–Wallis tests) were used for non-normally distributed variables.

Preoperative and postoperative clinical scores (Kujala, Lysholm, Tegner, and VAS) were compared using the paired samples *t*-test. Correlations between lesion size and postoperative functional scores were analyzed using Pearson’s correlation coefficient (r).

Because multiple subgroup analyses were performed, the risk of type I error was considered. Bonferroni correction was applied for all post hoc comparisons; however, given the exploratory nature of this study, no global adjustment was performed across independent analyses. This approach aligns with the primary aim of identifying clinically meaningful trends rather than establishing definitive causal relationships.

A priori power analysis was performed using G*Power version 3.1.9.7 (Heinrich Heine University, Düsseldorf, Germany) to determine the minimum required sample size. Based on preliminary data from a pilot cohort (effect size f = 0.35, α = 0.05, power (1–β) = 0.80) for detecting differences in postoperative Kujala scores between lesion localization groups (four groups, one-way ANOVA), the minimum sample size required was calculated as 63 patients. Considering potential data loss during follow-up, a total of 75 patients were included, which provided a post hoc power of 0.87, confirming adequate statistical strength for the primary outcome [16].

## 3. Results

A total of 75 patients (46 females, 29 males) who underwent osteochondral fixation following patellar dislocation met the inclusion criteria and were analyzed. The mean age of the cohort was 23.8 ± 6.1 years, representing a predominantly young and active patient population. The mean body mass index (BMI) was 24.6 ± 3.2 kg/m^2^. In terms of injury characteristics, the right knee was affected in 54.7% of cases, and traumatic mechanisms accounted for the majority of dislocations (77.3%). The average duration between the index dislocation and the surgical procedure was 3.8 ± 2.1 months. Among the total cohort, 29.3% required concurrent ligamentous procedures such as medial patellofemoral ligament (MPFL) reconstruction during the fixation surgery. These demographic and baseline clinical findings are summarized in Table 1, providing a general overview of the studied population.

Lesion distribution analysis demonstrated that osteochondral injuries were most frequently located on the medial patellar facet (42.7%). The trochlear groove/lateral femoral condyle was the second most common site (34.7%), followed by the lateral facet (12.0%) and multi-site lesions (10.7%). Significant morphological differences were identified among these anatomical regions. Lesions located at the trochlea and especially multi-site involvement exhibited markedly larger defect areas, greater lesion depth, and a higher rate of >2 mm fragment displacement when compared with medial or lateral facet lesions. These differences were statistically significant, as demonstrated by ANOVA and chi-square testing (lesion size: F = 4.92, *p* = 0.003; depth: F = 3.47, *p* = 0.021; displacement: χ^2^ = 7.84, *p* = 0.049). The complete distribution of lesion morphology is detailed in Table 2.

Surgical characteristics showed that metallic screws were the primary fixation material (56.0%), followed by bioabsorbable screws or darts (33.3%) and smooth pins (10.7%). The mean number of fixation devices used per case was 1.7 ± 0.8. Concomitant realignment procedures—either tibial tubercle transfer or lateral retinacular release—were performed in 24.0% of patients, reflecting the presence of associated patellar maltracking or instability. The mean operative duration was 72 ± 18 min. Intraoperative complications were infrequent, occurring in only 4.0% of the cases. These surgical details are summarized comprehensively in Table 3.

At the final 12-month follow-up, all functional outcome measures demonstrated statistically significant improvement compared with preoperative values. The mean Kujala score increased from 62.4 ± 11.3 to 86.7 ± 9.1, while the Lysholm score improved from 64.1 ± 12.0 to 88.2 ± 8.3 (both *p* < 0.001). Activity level, assessed via the Tegner scale, improved from 3.1 ± 1.0 to 5.0 ± 1.2 (*p* < 0.001). Pain levels measured by VAS decreased substantially from 6.2 ± 1.3 to 2.1 ± 1.1 (*p* < 0.001). Furthermore, 78.7% of patients successfully returned to their pre-injury sporting level within a mean of 8.9 ± 2.7 months. These functional outcomes are summarized in Table 4, reflecting a strong overall clinical response to fixation.

When outcomes were compared according to lesion morphology, variations in postoperative recovery became evident. Patients with medial facet lesions achieved the highest postoperative Kujala and lowest VAS pain scores, followed closely by lateral facet lesions. In contrast, trochlear and multi-site lesions demonstrated lower functional scores and higher pain levels at follow-up. Similarly, when stratified by lesion size, patients with defects <100 mm^2^ showed significantly better functional outcomes and lower pain scores compared with those with larger lesions (*p* = 0.001). These subgroup findings are presented in Table 5, highlighting the influence of both lesion localization and size on recovery parameters.

Postoperative complications were observed in a total of eight patients (10.7%). The most common complication was recurrent patellar dislocation (4.0%), followed by hardware irritation (2.7%), arthrofibrosis (2.7%), and radiographic nonunion of the osteochondral fragment (1.3%). All complications were managed successfully either conservatively or surgically, and no postoperative infections were recorded. Detailed information regarding complication types, timing, and management strategies is presented in Table 6.

## 4. Discussion

This study investigated the relationship between the localization and size of osteochondral lesions and postoperative clinical outcomes in patients who underwent fixation following patellar dislocation. The principal findings were that medial patellar facet involvement was the most frequent presentation; larger or deeper lesions, particularly those involving the trochlear groove or multiple regions, were associated with lower functional scores and higher residual pain levels. In addition, all patients demonstrated significant postoperative improvements in pain and functional outcomes following osteochondral fixation, regardless of implant type.

Acute lateral patellar dislocation induces substantial shear forces across the patellofemoral articulation, typically resulting in impaction between the medial patellar facet and the lateral femoral condyle. The magnitude and vector of the traumatic force determine the extent of osteochondral damage [3,22,23]. Medial facet lesions are generally more contained and less exposed to compressive stress, whereas trochlear and condylar lesions tend to be larger and subjected to complex multidirectional loading patterns during knee motion [24,25].

From a biomechanical standpoint, the patellofemoral joint experiences peak contact pressures during 30–60° of knee flexion, a range frequently involved during sports or stair climbing. Lesions located within this contact zone, particularly in the trochlear groove, may disrupt the congruence of the joint surface and alter load transmission, leading to increased contact stress and delayed cartilage repair [26,27]. These altered mechanical conditions may explain the relatively lower Kujala and Lysholm scores observed in patients with trochlear or multi-regional lesions in the present series.

Biologically, the osteochondral fragment’s viability depends on prompt anatomic reduction, stable fixation, and early revascularization from the subchondral bone. Delays in fixation or instability at the repair site can compromise subchondral perfusion, resulting in incomplete healing or fibrocartilaginous repair tissue. Previous studies have demonstrated that fragments smaller than approximately 100 mm^2^ show higher healing potential, whereas larger defects exhibit impaired load distribution and diminished functional recovery [28,29]. Our findings support this concept, as lesion size correlated inversely with functional improvement and directly with postoperative pain intensity.

The outcomes of this study are generally consistent with those reported in the literature, where osteochondral fixation following patellar dislocation has yielded pain relief and satisfactory function in 70–90% of cases [30,31,32]. In our cohort, Kujala and Lysholm scores significantly improved at the 12-month follow-up, while VAS pain levels decreased by more than 60%. A return-to-sport rate of 78.7% aligns with previous mid-term reports in the literature [33,34,35]. These results underscore the clinical efficacy of timely fixation and structured rehabilitation in restoring joint congruence and function.

Regarding fixation materials, no significant difference in outcomes was observed between metallic and bioabsorbable implants. This observation agrees with several comparative studies suggesting that implant type is less influential than accurate reduction, sufficient compression, and early mobilization [36]. Although bioabsorbable devices eliminate the need for hardware removal and reduce imaging artifacts, their biomechanical stability remains comparable to that of metallic fixation when properly applied.

Lesion localization and surface area appear to be critical prognostic determinants. Larger defects, especially those exceeding 100 mm^2^ or located within the trochlear groove, are exposed to greater mechanical stress during knee flexion and extension. These lesions can alter the kinematics of the patellofemoral joint, elevate focal contact pressures, and accelerate subchondral bone remodeling, all of which may adversely affect long-term clinical outcomes [37,38]. Conversely, smaller medial facet lesions tend to heal under relatively favorable mechanical conditions, with more uniform stress distribution and less motion at the repair interface.

The prognostic impact of lesion morphology is further supported by the intrinsic anatomical and histological differences across patellofemoral regions. Cartilage within the medial facet is generally thicker, more homogeneous, and more resistant to shear forces, whereas trochlear cartilage is thinner, more vulnerable to tangential loading, and located in a region that undergoes higher patellofemoral contact forces during early-to-mid flexion [10,39]. These structural variations provide a biologically plausible explanation for the superior outcomes observed in medial facet lesions and the relatively poorer healing observed in trochlear lesions in our cohort. Moreover, trochlear lesions often present with marginal fragmentation or irregular borders that complicate precise anatomic reduction and increase the risk of persistent step-off or incongruence, which has been strongly correlated with postoperative anterior knee pain in prior biomechanical studies [40,41].

Our findings also address a key gap in current evidence. Many previous studies investigating the role of lesion morphology relied on small sample sizes, focused on isolated lesion subtypes, or lacked adequate heterogeneity, leading to inconsistent correlations between lesion characteristics and prognosis [14,18,28]. In contrast, the present study incorporates a broader morphological spectrum—including patellar, trochlear, and multifocal lesions—and therefore provides more comprehensive and generalizable data supporting lesion morphology as a major prognostic determinant.

The observed decline in outcomes among lesions exceeding 100 mm^2^ suggests a clinically meaningful threshold that may guide preoperative planning. Although not a definitive cut-off, this dimension aligns with previously proposed limits beyond which structural stability, load distribution, and subchondral perfusion become progressively compromised. In lesions larger than this threshold, surgeons might consider adjunctive interventions—such as osteochondral autograft transfer, particulated juvenile cartilage, or scaffold-based augmentation—particularly for physically active patients or those with trochlear involvement. The identification of size-based risk zones may ultimately contribute to future treatment algorithms and personalized surgical decision-making.

The biological disadvantages of large and trochlear lesions may also contribute to delayed or incomplete recovery. Larger fragments exhibit slower revascularization, greater surface area requiring integration, and increased susceptibility to micro-motion at the bone–cartilage interface. Trochlear lesions, specifically, may coexist with subtle maltracking or early dysplastic features that are not always overt radiographically but still increase the mechanical load experienced during gait and stair activities [27,42]. These factors may explain the lower Kujala and Lysholm scores observed in patients with trochlear or multifocal lesions.

Rehabilitation may also modulate the relationship between lesion morphology and postoperative outcomes. Although all patients followed a standardized rehabilitation protocol, minor variations in adherence, quadriceps activation patterns, or neuromuscular control could disproportionately affect trochlear or larger lesions due to their higher mechanical sensitivity. Future studies incorporating objective strength measurements, gait analyses, or monitored rehabilitation compliance could improve understanding of this interaction.

Measurement reliability is essential when interpreting size-dependent outcomes. The strong interobserver agreement observed in our MRI-based measurements (ICC = 0.88) indicates that fragment size and depth were assessed reproducibly and consistently, minimizing the potential for measurement bias. The incorporation of reliability analysis enhances methodological rigor and underscores the importance of standardized MRI evaluation when assessing osteochondral lesion morphology.

The observed association between lesion size and postoperative functional outcomes highlights the importance of early detection and surgical intervention before the development of extensive chondral damage. In clinical practice, detailed preoperative imaging and careful intraoperative mapping of the lesion may guide fixation strategy, choice of implant, and rehabilitation planning.

The present findings emphasize that osteochondral fixation remains a reliable method for restoring articular congruity and knee function after patellar dislocation, provided that appropriate patient selection and anatomical reduction are achieved. However, surgeons should recognize that lesion morphology—particularly size and location—plays a substantial role in determining postoperative recovery. Identifying high-risk lesion patterns, such as large trochlear or multifocal defects, may allow for closer monitoring, individualized rehabilitation, or even consideration of adjunctive cartilage restoration techniques in selected cases.

MRI plays a pivotal role not only in diagnosing osteochondral injuries but also in assessing prognostic morphology. In this study, preoperative MRI enabled precise measurement of fragment size and depth, while T2 hyperintensity in the subchondral zone provided indirect evidence of bone marrow involvement. Previous studies have emphasized that persistent subchondral edema and irregular articular surface restoration predict slower cartilage recovery and residual anterior knee pain. Therefore, standardized MRI assessment may serve as a valuable adjunct for risk stratification and long-term monitoring following osteochondral fixation.

Several limitations must be acknowledged when interpreting the findings of this study. First, the retrospective nature of the analysis carries an inherent risk of selection bias and missing data, which may limit the ability to establish causal relationships between lesion morphology and clinical outcomes. Although strict inclusion criteria were applied, unmeasured confounders—such as baseline activity level, pain chronicity, or subtle patellofemoral malalignment—may still have influenced the results.

Second, the follow-up period of 12 months may not fully capture long-term cartilage remodeling, subchondral bone adaptation, or the eventual development of patellofemoral osteoarthritis, especially in patients with larger or trochlear lesions. Longer-term imaging and functional assessments would be valuable in determining whether the early advantages observed in medial facet lesions persist over time.

Third, lesion size and depth were measured using MRI rather than intraoperative quantitative mapping. Although interobserver reliability was excellent (ICC = 0.89), MRI-based assessment may still be affected by slice thickness, reconstruction variability, and cartilage–bone interface irregularities, potentially introducing measurement error. The use of advanced techniques such as 3D isotropic MRI or intraoperative optical mapping could improve accuracy in future studies.

Fourth, functional outcomes were assessed using patient-reported scoring systems, which—despite their clinical relevance—may be influenced by patient perception, motivation, or lifestyle changes. The absence of objective performance-based measures (e.g., isokinetic strength testing, gait analysis, hop tests) limits the ability to correlate lesion morphology with biomechanical recovery. Additionally, radiological union was evaluated qualitatively, without quantitative cartilage assessment such as T2 mapping or dGEMRIC, which could more precisely characterize healing quality.

Fifth, although all patients followed the same rehabilitation protocol, individual variation in adherence, neuromuscular control, quadriceps activation, and progression speed could have influenced recovery, particularly in higher-risk lesion groups. Rehabilitation heterogeneity is a common but unavoidable limitation in retrospective clinical studies.

Sixth, despite standardized surgical techniques, the procedures were performed by different surgeons, introducing potential operator-dependent variability in reduction quality, fixation compression, or adjunctive procedures such as MPFL reconstruction or tibial tubercle transfer. Implant choice (metallic vs. bioabsorbable) was also partly surgeon-dependent, which may contribute to subtle differences not captured statistically.

Finally, histologic confirmation of osteochondral fragment healing or cartilage integration was not feasible due to the clinical nature of the study. Advanced imaging or second-look arthroscopy could provide additional insight but was not ethically justified in this setting.

Despite these limitations, the study’s strengths include an adequate sample size supported by power analysis (*n* = 75, post hoc power = 0.87), homogeneous inclusion criteria, standardized postoperative rehabilitation, and the integrated evaluation of morphological and clinical parameters. These components together enhance the validity of the findings and contribute meaningful evidence regarding the prognostic role of lesion size and localization in osteochondral fixation after patellar dislocation.

## 5. Conclusions

In conclusion, lesion localization and size significantly influence postoperative outcomes following osteochondral fixation for patellar dislocation. Patients with medial facet lesions achieved better functional recovery compared with those with trochlear or larger lesions. Stable anatomical fixation and early rehabilitation resulted in overall satisfactory clinical improvement. These findings suggest that detailed morphological evaluation should be an integral part of surgical planning and prognostic counseling in the management of osteochondral injuries of the patellofemoral joint.

## Figures and Tables

**Table 1:** Baseline demographic and clinical characteristics (N = 75).

Variable	Mean ± SD or *n* (%)	*p*-Value
Age (years)	23.8 ± 6.1	—
Sex (Male/Female)	29 (38.7%)/46 (61.3%)	0.412
Affected side (Right/Left)	41 (54.7%)/34 (45.3%)	0.376
BMI (kg/m^2^)	24.6 ± 3.2	0.287
Time from dislocation to surgery (months)	3.8 ± 2.1	0.241
Mechanism (Traumatic/Atraumatic)	58 (77.3%)/17 (22.7%)	0.093
Prior dislocation episodes	2.3 ± 1.7	—
Concomitant ligamentous procedure	22 (29.3%)	0.128

BMI, body mass index; MPFL, medial patellofemoral ligament.

**Table 2:** Distribution and morphological characteristics of osteochondral lesions.

Localization	*n* (%)	Lesion Size (mm^2^, Mean ± SD)	Depth (mm, Mean ± SD)	Displacement >2 mm *n* (%)
Medial patellar facet	32 (42.7%)	95 ± 38	3.1 ± 1.0	9 (28.1%)
Lateral patellar facet	9 (12.0%)	88 ± 30	2.8 ± 0.9	2 (22.2%)
Trochlear groove/LFC	26 (34.7%)	130 ± 45	3.7 ± 1.2	11 (42.3%)
Multiple sites	8 (10.7%)	165 ± 60	4.0 ± 1.3	5 (62.5%)
Overall comparison	—	F = 4.92, *p* = 0.003	F = 3.47, *p* = 0.021	χ^2^ = 7.84, *p* = 0.049

Post hoc (Tukey): Multiple > Medial (*p* = 0.012), Multiple > Lateral (*p* = 0.004), Trochlear > Medial (*p* = 0.038) LFC, lateral femoral condyle; SD, standard deviation; ANOVA, analysis of variance.

**Table 3:** Surgical parameters and fixation techniques.

Parameter	Value	*p*-Value
Metallic screw	42 (56.0%)	—
Bioabsorbable screw/dart	25 (33.3%)	—
Smooth pin/K-wire	8 (10.7%)	—
Number of fixation devices	1.7 ± 0.8	0.214
Concomitant realignment	18 (24.0%)	0.142
Operative time (minutes)	72 ± 18	0.317
Intraoperative complications	3 (4.0%)	—

**Table 4:** Functional outcomes and clinical scores.

Outcome	Preoperative	Postoperative	*p*-Value
Kujala score	62.4 ± 11.3	86.7 ± 9.1	<0.001
Lysholm score	64.1 ± 12.0	88.2 ± 8.3	<0.001
Tegner activity level	3.1 ± 1.0	5.0 ± 1.2	<0.001
VAS pain	6.2 ± 1.3	2.1 ± 1.1	<0.001
Return to sport	—	59 (78.7%)	—
Time to return (months)	—	8.9 ± 2.7	—

VAS, visual analogue scale.

**Table 5:** Relationship between lesion morphology and postoperative outcomes.

Variable	Kujala Score (Mean ± SD)	VAS Pain	Comparison	*p*-Value
**Lesion localization**	—	—	ANOVA	0.02
• Medial facet (*n* = 32)	88.6 ± 8.1	1.9 ± 1.0	—	—
• Lateral facet (*n* = 9)	87.9 ± 8.5	2.0 ± 0.9	—	—
• Trochlear/LFC (*n* = 26)	83.2 ± 9.6	2.4 ± 1.1	—	—
• Multiple sites (*n* = 8)	80.1 ± 10.2	2.7 ± 1.2	—	—
Lesion size (<100 vs. ≥100 mm^2^)	88.9 ± 8.2/82.7 ± 9.5	1.9 ± 0.9/2.5 ± 1.1	*t*-test	0.001

VAS, visual analogue scale; LFC, lateral femoral condyle.

**Table 6:** Postoperative complications.

Complication	*n* (%)	Time to Event (Months)	Management
Recurrent dislocation	3 (4.0%)	7.3 ± 2.5	Revision MPFL reconstruction
Hardware irritation	2 (2.7%)	4.1 ± 1.2	Implant removal
Arthrofibrosis	2 (2.7%)	3.8 ± 1.0	Manipulation under anesthesia
Nonunion	1 (1.3%)	5.5	Conservative treatment
Total	8 (10.7%)	—	—

MPFL, medial patellofemoral ligament.

## Data Availability

The data presented in this study are available on reasonable request from the corresponding author. The data are not publicly available due to ethical and privacy restrictions.

## Abbreviations

ANOVA	Analysis of Variance
BMI	Body Mass Index
K-wire	Kirschner Wire
LFC	Lateral Femoral Condyle
MRI	Magnetic Resonance Imaging
MPFL	Medial Patellofemoral Ligament
SD	Standard Deviation
SPSS	Statistical Package for the Social Sciences
TTT	Tibial Tubercle Transfer
VAS	Visual Analogue Scale
